# The Job Satisfaction of Finnish Nursing Staff: The Development of a Job Satisfaction Scale and Survey Results

**DOI:** 10.1155/2012/210509

**Published:** 2012-10-23

**Authors:** Tarja Kvist, Raija Mäntynen, Pirjo Partanen, Hannele Turunen, Merja Miettinen, Katri Vehviläinen-Julkunen

**Affiliations:** ^1^Department of Nursing Science, University of Eastern Finland, Kuopio Campus, P.O. Box 1627, 70211 Kuopio, Finland; ^2^Kuopio University Hospital, P.O. Box 1777, 70211 Kuopio, Finland; ^3^University of Tampere, 33014 Tampere, Finland

## Abstract

This paper describes the development of the Kuopio University Hospital Job Satisfaction Scale (KUHJSS) and the results of the survey. The scale was developed through a systematic literature review, and its validity and reliability were assessed using several psychometric properties including expert evaluation (*n* = 5), a pilot survey (*n* = 172), and exploratory factor analysis. The final version of KUHJSS included 37 items. A large sample psychometric evaluation was made by nursing staff (*n* = 2708). The exploratory factor analysis revealed seven factors with modest internal consistency (0.64–0.92). The staff reported relatively high job satisfaction. The greatest satisfaction was derived from motivating factors associated with the work; the least, from the job's demands. Respondents who considered their working units to provide an excellent quality of care reported the highest job satisfaction in every subarea (*P* < .0001). The KUHJSS proved to be a reliable and valid tool for measuring job satisfaction in hospital care.

## 1. Introduction

Job satisfaction has been described as the extent to which employees like their jobs [1–3]. It can be further defined in relation to employees' expectations of their work. Unless these expectations are fulfilled, employees may be dissatisfied with their jobs [4]. Job satisfaction is described in terms of psychological concepts, including attitudes towards work, work ethic, professional development, the development of work, and even, to some extent, perceptions of the meaning of life [5, 6]. Job satisfaction is an emotional state that is attained on achieving the results at which the individual is aiming [7]. Working conditions also have an effect on employees' well-being [3, 8–10].

One element of job satisfaction is the positive experience of being a part of a well-functioning work group [4, 9, 11–13]. Job satisfaction encompasses a range of elements affected both by factors internal to the work place and employees' attitudes and behavior, as the concept of job satisfaction is shaped by each staff member's personal perspective [14].

Recruiting new staff while maintaining a strong commitment to the organization among current staff is a significant challenge for the health care sector. In the future, it will be necessary to prioritize job satisfaction in health care if hospitals are to ensure adequate staffing by maintaining high levels of commitment amongst existing employees while recruiting new staff [6, 8, 15–19]. Several studies have shown a significant positive correlation between the job satisfaction of nursing staff and their commitment to their work [6, 20, 21].

An important predictor of job satisfaction is the quality of collaboration between nursing staff members [22] and between nursing staff and nurse managers [1, 3, 23]. In a number of studies, nursing staff expressed the desire to receive more feedback, more understanding, and fairer treatment from their nurse manager. It appears that job satisfaction among nursing staff can be significantly increased by the adoption of a supportive leadership style that incorporates effective communication, psychological rewards, feedback, support, recognition, and mutually defined goals [3, 10, 24–31]. 

Job satisfaction has a positive effect on quality of care and patient outcomes. The best quality of care is realized where nursing staff rate their job satisfaction highly. Hospitals with high job satisfaction scores have been reported to also have high quality of care and favorable patient outcomes [4, 14, 32, 33]. 

In a literature review, the Finnish researchers, Utriainen and Kyngäs [34], found that most studies on job satisfaction among hospital nurses have been conducted using different methods and instruments [1, 33, 35], most of which were developed in the United States. The most well-known instruments are the Index of Work Satisfaction [36], the McCloskey and Mueller Satisfaction Scale [37], the Nursing Work Index [35, 38], the Nursing Work Index-Revised [39], the Practice Environment Scale-Nursing Work Index [40], and the Perceived Nursing Work Environment [41]. In another review, Hall [35] notes that instruments such as the Index of Work Satisfaction and McCloskey and Mueller Satisfaction Scale have been well developed and widely used, and that their reliability has been evaluated in different research environments. However, more attention should be paid to the validity of these instruments. There is a need to accurately determine the nature of the relationship between job satisfaction among nursing staff and nurse-sensitive patient outcomes. 

Although several useful instruments exist, there is a need for culturally sensitive instruments in health care systems outside the United States that may be organized in very different ways. In particular, the most widely used existing instruments focus on the nurses' working environment in general rather than job satisfaction specifically [39, 40]. The Finnish researcher group of At Safe Hospital Project set a goal to develop a scale for measuring the job satisfaction of hospital nursing staff and even job satisfaction of whole hospital staff including all staff working at the hospital. The aim was to develop both a reliable and culturally sensitive instrument. The whole hospital nursing staff was considered in this study including four groups: nurse leaders (nursing directors, nurse managers), registered nursing staff (registered nurses, midwives, radiographers, or laboratory nurses), practical nurses, and other workers. In Finland all these groups are called nursing staff. They all take care of patients in different phases of patients' paths in hospital. 

The majority of Finnish health care services are organized and provided by the municipal health care system. Municipalities are legally required to provide adequate health services for their residents. The services are funded by taxes, and the municipalities receive state subsidies. Specialized health care in the municipal system is provided by 20 hospital districts, each of which is owned and funded by its member municipalities. Each hospital district has one or several hospitals, one of which is a central hospital, and five hospital districts have a university hospital [42]. At Safe Hospital Project began in 2006 in the Northern Savo Hospital District, where one university hospital and three central hospitals with around 2500 beds and 6,000 nursing staff serve a local population of 860,000.

## 2. Aims of the Study

The aims of this study were to

1. develop a scale for assessing the job satisfaction of nursing staff and describe the process by which this was accomplished,
2. assess the job satisfaction of nursing staff using the newly-developed scale,
3. identify differences in the job satisfaction of nursing staff in relation to demographic variables.

## 3. Methods

### 3.1. Phase 1: Item Selection for the Job Satisfaction Scale

The first step in the development of the scale involved conducting a systematic literature review and using the results obtained to define and analyze the concept of job satisfaction. This work was conducted in 2007 on the basis of searches of Medline (2000–2007), CINAHL (2000–2007), and the Finnish databases Kuopus (2000–2007) and Arto (2000–2007). We used the key words “job satisfaction,” “healthcare personnel,” “work satisfaction,” “nursing staff,” and “nurse.” The Boolean operators AND and OR were used in the search. The languages used were English and Finnish. Twenty-three study papers were selected after reviewing the titles, abstracts, and full texts of the hits obtained (Table 1). All of the selected studies described the job satisfaction of the nursing staff from different perspectives and had been published in well-regarded journals; in every case, the design and context of each study was well explained and the reliability of the results reported and conclusions drawn was high. 

On the basis of the literature review, 16 demographic variables and 53 items related to job satisfaction were identified. The content analysis of these items revealed five subareas of job satisfaction (Table 1). The content validity of the Job Satisfaction Scale was evaluated by five staff nurses working at the university hospital. Members of the research group also reviewed and made changes to the items on a number of occasions, including after a pilot test involving five nurses. These members of the research group (expert panel) were working or closely collaborated with the university hospital. They included an MNSc student (the RN); a post-doctoral researcher (PhD) working as a clinical researcher in both the university and university hospital; another postdoctoral researcher (PhD) who was teaching at the university, and collaborating with the university hospital and had experience as a RN and nurse manager in the university hospital; the Chief Nursing Officer of the University Hospital (PhD); a professor of Nursing Science, who was partly working at the university hospital, and she had the expertise in the development of the instruments.

## 4. Results of the Development of the Job Satisfaction Scale

### 4.1. Phase 2: Feasibility and Preliminary Psychometric Evaluation

It was important to frame each question so that it addressed only a single issue [45]. At the pilot stage, the Job Satisfaction Scale included 53 items relating to five different subareas as well as items relating to 16 demographic variables; this number was later reduced. 

A test item was created for each 53 items, and respondents were asked to answer each item on the test using a five-point Likert scale. The possible responses to each item were 1 = strongly disagree; 2 = partly disagree; 3 = neither agree nor disagree; 4 = partly agree; 5 = strongly agree. 

 A pilot survey of total 503 nursing staff in medical areas of four study hospitals was conducted using a questionnaire; this resulted in a total of 172 responses, giving a response rate of 34%. The construct validity of the Job Satisfaction Scale with 53 items (Table 1) was evaluated using exploratory factor analysis, with the aim of reducing the number of items in the scale and identifying any underlying latent variables.

Principal axis factoring was applied, because a theoretical underlying factor structure was expected on the basis of the systematic literature review. The rotation technique applied was the varimax method with Kaiser normalization. In varimax rotation, the factors are rotated for the best factor solution, which was the aim in this study. The Kaiser-Meyer-Olkin test value for the responses was good (0.80), and the Bartlett's test of sphericity result was highly significant (*P* < 0.001); thereby confirming that factor analysis was appropriate for these items. This factor solution had 39 items, all of which met the correlate over 0.3 at least with one item and they loaded of over 0.3. A cut-off point 0.3 is generally selected for the correlations and loadings, as used in this study, too. The loadings over 0.3 show us the items will be included as elements of the factor [45].

The exploratory factor analysis resulted in seven factors describing nurses' job satisfaction. The results obtained in the factor analysis demonstrated the soundness of the original instrument's theoretical structure.

The reliability of the Job Satisfaction Scale was assessed by evaluating its internal consistency using Cronbach's alpha. The Cronbach's alpha values for the scale ranged from 0.72 to 0.89, indicating that the new instrument has only modest degree of internal consistency [46, 47].

### 4.2. Phase 3: Large Sample Psychometric Evaluation

In addition to the internal consistency demonstrated by the Cronbach's alpha values, the scale's reliability was also evaluated by applying it to a large number of subjects. In order for the results of a factor analysis to be reliable, it should be conducted on a dataset obtained from a sample containing at least five subjects per item on the scale, according to Watson and Thompson [48], and perhaps as many as ten [45]. These criteria were not satisfied in the pilot study, which had 172 respondents. However, it did meet the criterion suggested by some statisticians that two subjects per item on the scale are sufficient [49]. A large sample psychometric evaluation of the Job Satisfaction Scale was performed by gathering responses from the entire nursing staff of one university hospital and three central hospitals (*N* = 5778) in Finland's Northern Savo hospital district in 2008. The questionnaire took the form of a web-based survey in three of the hospitals and a mailed questionnaire in one hospital. A total of 2708 responses were received, giving a response rate of 47%. In the latter phase the applied scale was named the Kuopio University Hospital Job Satisfaction Scale (KUHJSS). 

Before applying exploratory factor analysis to the data for the 2708 respondents, two items were removed from KUHJSS by the expert panel, one because its content overlapped with a different item, and the other because its content was deemed inappropriate. The final factor analysis thus included 37 items. The responses were subjected to factor analysis using the principal axis method; rotation was accomplished using the Varimax method with Kaiser Normalization. The Kaiser-Meyer-Olkin test value for the responses was 0.94, indicating a sufficiently high degree of correlation between the items, as did Bartlett's test of sphericity (*P* < 0.001). The correlation matrix was examined; it was found that every item correlated with at least one other item with a correlation coefficient in excess of 0.3. Only one item had the loading slightly below 0.3 it was retained because it measured important aspect of job satisfaction. All of the factors' eigenvalues were greater than one, and they accounted for 46.4% of the observed variance (2.9–13.9). On the basis of this exploratory factor analysis, seven factors were identified: leadership (7 items); requiring factors of work (8 items); motivating factors of the work (6 items); working environment (4 items); working welfare (4 items); participation in decision-making (4 items); sense of community (4 items) (Table 2). The final factor solution differed from the pilot solution; only the leadership factor retained all of the original items. These changes improved the construct validity of the final questionnaire since they were made on the basis of a more reliable factor analysis; the second factor analysis was more credible than the first because it was performed using responses from a much larger pool of participants such that there were more than five subjects per item [45, 48]. The reliability of the scale was only modest, as indicated by its internal consistency; the Cronbach alphas of the mean scores ranged from 0.64 to 0.92 (Table 2).

### 4.3. Phase 4: Application of KUHJSS

#### 4.3.1. Data Analysis

Research data were analyzed using SPSS 14.0 for Windows [50]. Frequencies and percentage distributions were used to summarize the raw data. The final version of the instrument examines 11 demographic variables: hospital, gender, age, working unit, working division, work position, working experience (length of time) in current unit, overall work experience (length of time), type of employment, working hours, and the quality of care in the working unit. Five demographic variables were disregarded, because respondents declined to answer questions relating to them in more than half of all cases in a pilot study and they did not seem to be significantly related with the job satisfaction of nurses.

Different categorization schemes were used to group the responses to items relating to specific demographic variables. These categorization schemes have been found to be practical and reliable [43]. Four respondent age groups were defined: group 1 = under 30 years, group 2 = 31–40 years, group 3 = 41–50 years, and group 4 = over 51 years. Five groups were defined for respondents' years of work experience in the current work unit and total years of work experience: group 1 = no more than one year, group 2 = 2–5 years, group 3 = 6–10 years, group 4 = 11–20 years, group 5 = over 21 years. The respondents evaluated the quality of care in the working unit in accordance with Finnish school grades, with scores ranging from 4 (worst) to 10 (best). This variable was used to classify the working units into three groups: group 1 (poor quality) = 4–6, 2 (moderate) = 7-8, and 3 (excellent) = 9-10. On the basis of the exploratory factor analysis, seven mean scores were calculated, one for each subarea. The possible mean scores ranged from 1 (lowest) to 5 (highest); they were calculated by summing the values of the respondents' answers to all of the items in the relevant subarea and dividing the result by the number of items. The normality of the distributions of these mean scores was tested using the Kolmogorov-Smirnov test. Since many of the mean scores were not normally distributed, nonparametric tests (specifically, the Mann-Whitney *U*-test and the Kruskall-Wallis test) were used for data analysis (Table 3).

#### 4.3.2. Demographic Information on the Nursing Staff

The job satisfaction questionnaire was returned by 2708 members of the nursing staff. The majority of them were female (89%). The average age of the respondents was 43; the youngest was 19, and the oldest was 67. Of these respondents, 71% were registered nurses, midwives, radiographers, or laboratory nurses (all of whom perform demanding professional nursing work). On average, the respondents had worked in their current units for approximately ten years and had 18 years' work experience in total. Eighty-one percent of the nursing staff members were permanent employees, and 64% of them worked on shifts. The average evaluation of the quality of care provided by the respondents' working units was 8.11, which is moderate.

#### 4.3.3. Nursing Staff Evaluations of Their Job Satisfaction

Overall, the nursing staff reported relatively high job satisfaction (mean 3.59). The greatest degree of satisfaction was derived from the motivating factors of the work (mean 4.18). Considerable satisfaction was also derived from working welfare (mean 4.12), leadership (mean 3.67), and sense of community (mean 3.67); the lowest degree of satisfaction related to requiring factors of work (mean 3.04).

#### 4.3.4. Factors Related to Job Satisfaction

Some demographic variables were related to the nursing staff's evaluation of their overall job satisfaction. These variables were the nurse's age, occupational group, and total work experience, as well as the identity of the hospital at which they worked, and whether they worked in shifts or not. One of the hospitals had lower job satisfaction than the three others in all subareas (*P* = .033 to *P* < .0001). The highest job satisfaction levels were reported by nurses over 51 years of age, nursing leaders, nurses with less than one year or more than 21 years of total work experience, and day shift workers (Table 3).

Males derived more satisfaction from leadership (*P* = .025) and sense of community (*P* = .025) than did females, while females rated the motivating factors of work (*P* < .0001) and participation in decision making (*P* = .001) more highly than males (Table 3).

Temporary employees were more satisfied with the requiring factors of the work than permanent employees (*P* = .026). Permanent employees rated motivating factors more positively than temporary working nursing staff (*P* = .016) (Table 3).

#### 4.3.5. The Relationship between Quality of Care and Job Satisfaction

The nurses' assessments of the quality of care provided by their working unit had a strong effect on their job satisfaction. Individuals who considered their units to provide an excellent quality of care also reported the highest degree of job satisfaction in every subarea (*P* < .0001) (Table 3).

## 5. Discussion

The first objective of this study was to develop a Job Satisfaction Scale for specialized health care nursing staff and to describe the process by which this was accomplished. The second was to use the newly developed scale to assess the job satisfaction of nursing staff, and the third was to identify relationships between background variables and nurses' job satisfaction. Although a number of studies on job satisfaction among nurses have previously been conducted (e.g., [1, 33, 34]) and a wide range of scales have been developed in recent decades [30, 39], developing of a Job Satisfaction Scale was useful in At Safe Hospital Project and it is giving reliable measurement and results for further research and development work.

### 5.1. The Development of Kuopio University Hospital Job Satisfaction Scale

The KUHJSS was developed in four phases according to instrument development guidelines. The first version of the scale included 53 items. After a pilot study and psychometric testing, a final version of KUHJSS was established that includes 37 items covering seven factors. These seven factors are leadership (7 items); requiring factors of work (8 items); motivating factors of the work (6 items); working environment (4 items); working welfare (4 items); participation in decision-making (4 items); sense of community (4 items). The KUHJSS contains some new items (e.g., My manager provides the staff feedback with the aim of developing work, items relating to motivating factors of the work) that are not so clearly present in earlier instruments that were mainly developed in the United States [34, 36–41].

The validity of the KUHJSS was evaluated by a number of methods, including expert review, a pilot study, and exploratory factor analysis. The structure of the scale was shown to be valid, and the subareas developed are coherent with respect to their content. Cronbach alpha values indicated that the instrument was internally consistent and therefore credible.

In future the validity and reliability of KUHJSS will be evaluated more extensively, for example, by using confirmatory factor analysis. It would also be useful to recode the answers to an alternative instrument developed in the US to investigate their convergence or divergence.

### 5.2. The Survey Results

The number of the respondents (*n* = 172) of the pilot study was not satisfactory. The web-based questionnaire was sent to 503 nursing staff, but in part because this web-based method was new and unfamiliar to them, we did not obtain as large a dataset as we had expected. In an earlier study using the traditional postal questionnaire, the response rates among nursing staff were much higher (up to 60%) [43]. However, over the course of a year we publicized the web-based method more widely and in 2008 we obtained a much higher number of respondents (*n* = 2708) for the survey, who provided sufficient data collected to allow both testing of the KUHJSS and generalization of the results within the hospitals surveyed.

The results obtained using the KUHJSS are useful for the image of nursing. The key result was that the nursing is very motivating work, which is a very important result when persuading young people to consider the nursing as a career. This is consistent with the findings of Fletcher [8] and indicates that nursing provides interesting and challenging work in which nurses can apply a wide range of skills and expertise and that nurses' motivation is enhanced by patient feedback. Nurses were satisfied with their working welfare. They look after their personal well-being and health, and are competent employees who actively strive for professional development. A high job satisfaction level among nursing staff is an important characteristic of attractive, “magnet” hospitals [3, 33]. According to this study, the job satisfaction levels of nursing staff were high in general, although some earlier studies have shown that nursing staff were not satisfied with their working conditions [4, 5, 14, 21].

However, nursing is also very demanding. The lowest job satisfaction scores were related to the requiring factors of the work. Previous studies conducted in Finland [43, 44] and elsewhere [4, 20, 21] identified low staffing levels as an important factor associated with low job satisfaction.

The nurses' reported job satisfaction also reflected their lack of opportunities to participate in decision-making; this is due to the bureaucracy of the Finnish health care system [42]. Shared governance relating to nursing practice and outcomes is not widespread in Finland [51]. In future, nurses and nurse leaders should serve on relevant councils and committees, with decision-making and information flowing bidirectionally and horizontally [52]. The results of this survey demonstrate that the nursing leadership in Finland will have to evolve and take on a transformational role if general job satisfaction is to be raised to the levels found in “magnet” hospitals [3, 51].

Nursing leaders reported the highest job satisfaction of all nurses who responded. The most satisfied nursing staff members were those who considered the quality of care provided by their working unit to be excellent. This relationship between job satisfaction and quality of care has been observed in previous studies [4, 8, 14, 32, 43].

## 6. Conclusions

The objective of developing a reliable and valid Job Satisfaction Scale was achieved, and a 37-item KUHJSS was developed. Responses to the scale provide an accurate and realistic picture of job satisfaction among Finnish nursing staff. Nursing staff are generally satisfied with their jobs, especially in terms of their motivation for work and their welfare at work. Job satisfaction was clearly related to nurses' perception of the quality of care provided by their working unit. However, nursing leaders should pay more attention to nurse staffing levels and to providing opportunities for nurses to combine their work and personal lives.

## Figures and Tables

**Table 1:** Job satisfaction subareas and items identified on the basis of the literature review (*the item is in KUHJSS in 2008).

Subarea number of items (*n*)	Items	Researcher and year
Demographic variables (16)	Gender*, age*, living status, number of children, taking care of elderly relatives, work position*, unit type, working unit*, working experience in current unit*, overall work experience*, type of employment*, working time type, working hours*, quality of care of last shift in working unit, quality of care in working unit generally*, changes of quality of care in last year.	Studies of this literature review

Leadership (8)	My manager is genuinely interested in the well-being of the staff.* My manager promotes the functional use of the work premises. My manager treats the staff fairly and equally.*	Kovner et al., 2006 [26]; Kvist, 2004 [43]; Ruggiero, 2005 [17]
	My manager provides the staff feedback with the aim of developing work.* My manager informs me thoroughly about issues concerning my unit.* My manager enables the continuous professional development of the staff.* My manager encourages staff to take part in the planning of our unit's operation.*	Fletcher, 2001 [8]; Kovner et al., 2006 [26]; Larrabee et al., 2003 [20]; Manojlovich and Laschinger , 2002 [14]; Rambur et al., 2005 [10]; Ruggiero, 2005 [17]; Sveinsdóttir et al., 2006 [27]; Upenieks, 2002 [24]
	My manager is interested in work results and outcomes.*	Rambur et al., 2005 [10]

Working unit (18)	My work unit is safe and secure.* My work unit is comfortable.* The workload is distributed evenly in my unit.*	Manojlovich and Laschinger, 2002 [14]; Roberts et al., 2004 [15]; Ruggiero, 2005 [17]
	I am satisfied with the planned work shifts.* I plan my work shift according to the principles of my unit. My unit allows the individuality with the work shift. It is possible to change the work shifts flexible in my unit. The work planning of my unit makes the every third weekend free.	Manojlovich and Laschinger, 2002 [14]; Ruggiero, 2005 [17]; Stordeur et al., 2007 [29]
	My unit is usually enough staffed.* The ratio between nurses and practical nurses is proper in my unit. New employees are familiarized well in my unit.* New employees are received welcome in my unit.	Cowin, 2002 [6]; Fletcher, 2001 [8]; Roberts et al., 2004 [15]; Upenieks, 2002 [24]
	Quality of care is high in my unit. Nursing models of my unit have been standardized based on the research evidence.	Kvist, 2004 [43]; Upenieks, 2002 [24]
	I trust the expertise of my colleagues.* There is a good community spirit in my unit.* The flow of information works well between nursing staff in my unit.* The flow of information works well between different staff groups in my unit.	Adams and Bond, 2000 [11]; Amos et al., 2005 [12]; Best and Thurston, 2004 [4]; Chu et al., 2003 [9]; DiMeglio et al., 2005 [13]; Fletcher, 2001 [8]; Kovner et al., 2006 [26]; Kvist, 2004 [43]; Larrabee et al. , 2003 [20]; Sveinsdóttir et al., 2006 [27]

Content and amount of work (8)	My work load is appropriate.*	Best and Thurston, 2004 [4]; Larrabee et al., 2003 [20]; Partanen, 2002 [44]; Shaver and Lacey, 2003 [21]
	I have opportunities to plan my nursing work independently.* I have opportunities to make independent decisions in my work.*	Larrabee et al., 2003 [20]; Manojlovich and Laschinger, 2002 [14]; Ma et al., 2003 [32]; Roberts et al., 2004 [15]; Ruggiero, 2005 [17]
	My work tasks are suitably challenging.* I can apply a wide range of my skills and expertise in my work.*	
	Client feedback motivates me in my work.* Challenging work motivates me. My work is interesting.*	Fletcher, 2001 [8]

Work organization (10)	The upper management of organization appreciates my work.* I have enough time for the high quality care. My organization has appropriate equipment for high quality patient care.*	Fletcher, 2001 [8]; Shaver and Lacey, 2003 [21]
	I am satisfied with my salary.*	Aiken et al., 2001 [5]; Amos et al., 2005 [12]; Cowin, 2002 [6]; Kovner et al., 2006 [26]; Kvist, 2004 [43]; Larrabee et al., 2003 [20]; Manojlovich and Laschinger, 2002 [14]; Rambur et al., 2005 [10]; Sveinsdóttir et al., 2006 [27]
	I have a chance to influence decision-making in my organization.*	Larrabee et al., 2003 [20]; Manojlovich and Laschinger, 2002 [14]; Upenieks, 2002 [24]
	My organization has appropriate work facilities.* My organization has well working occupational health care.	Fletcher, 2001 [8]; Manojlovich and Laschinger, 2002 [14]; Kvist, 2004 [43]; Manojlovich, 2005 [16]
	I have a chance of career development in the hospital district.*	Best and Thurston, 2004 [4]; Cowin, 2002 [6]; Sveinsdóttir et al., 2006 [27]
	Nursing of my organization is developed to be evidence based.	Upenieks, 2002 [24]
	I am willing to work in this hospital district in the future.*	Shaver and Lacey, 2003 [21]

Personal factors (9)	I appreciate my own work.* Combining work and personal life is successful.* Combining work and studying is successful.	Cowin, 2002 [6]; Rambur et al., 2005 [10]; Tourangeau and Cranley, 2006 [28]; Kovner et al., 2006 [26]; Stordeur et al., 2007 [29]
	I am active in developing myself professionally.* My stress tolerance is good. I do not find my work too stressful.* I feel I am a competent employee.* I look after my personal well-being.* I am happy with my current health.*	Larrabee et al., 2003 [20]; Manojlovich, 2005 [16]; Rambur et al., 2005 [10]; Ruggiero, 2005 [17]

**Table 2:** Results of exploratory factor analyses of KUHJSS (*n* = 2708) in 2008 (loadings, eigenvalues, Cronbach's alpha values).

Factor Items	Loading	Eigen values	Cronbach's alpha value
(1) Leadership		10.32	0.92
My manager/director is genuinely interested in the well-being of the staff.	0.82		
My manager/director treats the staff fairly and equally.	0.78		
My manager/director encourages staff to take part in the planning of our unit's operation.	0.77		
My manager/director provides the staff feedback with the aim of developing work.	0.76		
My manager/director informs me thoroughly about issues concerning my unit.	0.73		
My manager/director enables the continuous professional development of the staff.	0.71		
My manager/director is interested in work results and outcomes.	0.64		

(2) Requiring factors of work		3.11	0.82
My work load is appropriate.	0.69		
There are usually enough staff in my unit.	0.59		
I do not find my work too stressful.	0.56		
I am satisfied with my working hours.	0.52		
Combining work and personal life is successful.	0.52		
The workload is distributed evenly in my unit.	0.45		
My salary is appropriate in relation to the demands of my work.	0.43		
The upper management of the hospital district appreciates my work.	0.41		

(3) Motivating factors of the work		2.13	0.77
My work is interesting.	0.74		
I appreciate my own work.	0.65		
I can apply a wide range of my skills and expertise in my work.	0.52		
My work tasks are suitably challenging.	0.50		
Client feedback motivates me in my work.	0.43		
I am willing to work in this hospital district in the future.	0.41		

(4) Working environment		1.67	0.73
My unit has appropriate work facilities.	0.71		
My unit has appropriate equipment to ensure quality of care.	0.55		
My work unit is comfortable.	0.53		
My work unit is safe and secure.	0.39		

(5) Working welfare		1.37	0.64
I look after my personal well-being.	0.72		
I am happy with my current health.	0.59		
I am active in developing myself professionally.	0.43		
I feel I am a competent employee.	0.42		

(6) Participation in decision-making		1.19	0.75
I have opportunities to make independent decisions in my work.	0.68		
I have opportunities to plan my work independently.	0.57		
I have a chance to influence decision-making in my unit.	0.45		
I have a chance of career development in the hospital district.	0.29		

(7) Sense of community		1.08	0.69
I trust the expertise of my colleagues.	0.52		
There is a good community spirit in my unit.	0.49		
The flow of information works well in my unit.	0.41		
New employees are welcomed in my unit.	0.32		

**Table 3:** Relationships between demographic variables and job satisfaction (*N* = 2708).

Background variable	1 mean *P *	2 mean *P *	3 mean *P *	4 mean *P *	5 mean *P *	6 mean *P *	7 mean *P *	Total mean *P *
Hospital (*n* = 2707)								
Hospital 1 (*n* = 1176)	3.75	3.04	4.23	3.28	4.14	3.34	3.70	3.64
Hospital 2 (*n* = 340)	3.77	3.13	4.24	3.48	4.12	3.35	3.75	3.69
Hospital 3 (*n* = 410)	3.80	3.17	4.33	3.43	4.19	3.34	3.75	3.72
Hospital 4 (*n* = 781)	3.45	2.92	3.99	3.30	4.05	3.21	3.51	3.39
	<.0001	<.0001	<.0001	<.0001	.001	.033	<.0001	<.0001
Gender (*n* = 2671)								
Female (*n* = 2386)	3.66	3.03	4.21	3.34	4.12	3.33	3.66	3.59
Male (*n* = 285)	3.78	3.07	3.92	3.30	4.12	3.13	3.75	3.54
	.025	ns	<.0001	ns	ns	.001	.025	ns
Age (*n* = 2643)								
<30 years (*n* = 410)	3.66	2.95	4.11	3.34	4.10	3.36	3.70	3.56
31–40 years (*n* = 612)	3.60	2.95	4.09	3.31	4.10	3.26	3.60	3.52
41–50 years (*n* = 887)	3.63	3.01	4.18	3.25	4.13	3.26	3.62	3.56
>51 years (*n* = 734)	3.83	3.20	4.29	3.47	4.15	3.39	3.78	3.73
	<.0001	<.0001	<.0001	<.0001	ns	.016	<.0001	<.0001
Occupational group (*n* = 2708)								
Nurse leaders (*n* = 179)	3.89	3.52	4.38	3.77	4.35	4.17	4.14	3.97
Registered nursing staff (*n* = 1917)	3.61	2.98	4.17	3.31	4.12	3.26	3.61	3.54
Practical nurses (*n* = 499)	3.83	3.05	4.14	3.26	4.05	3.17	3.71	3.59
Others (*n* = 113)	3.74	3.25	4.15	3.54	4.12	3.42	3.71	3.70
	<.0001	<.0001	<.0001	<.0001	<.0001	<.0001	<.0001	<.0001
Work experience in current unit (*n* = 2660)								
<1 year (*n* = 426)	3.78	3.21	4.11	3.43	4.13	3.42	3.78	3.63
2–5 years (*n* = 743)	3.69	3.07	4.12	3.35	4.16	3.31	3.69	3.58
6–10 years (*n* = 526)	3.63	2.96	4.21	3.31	4.11	3.30	3.60	3.57
11–20 years (*n* = 599)	3.63	2.96	4.22	3.27	4.14	3.24	3.63	3.57
>21 years (*n* = 366)	3.65	3.05	4.24	3.41	4.05	3.29	3.65	3.60
	ns	<.0001	ns	.047	ns	ns	.008	ns
Work experience total (*n* = 2606)								
<1 year (*n* = 81)	3.92	3.06	4.22	3.47	3.97	3.52	3.85	3.68
2–5 years (*n* = 362)	3.66	3.05	4.13	3.37	4.13	3.38	3.68	3.58
6–10 years (*n* = 473)	3.58	2.91	4.05	3.30	4.09	3.21	3.59	3.48
11–20 years (*n* = 663)	3.64	2.99	4.17	3.27	4.15	3.30	3.62	3.56
>21 years (*n* = 1063)	3.73	3.12	4.26	3.38	4.13	3.34	3.72	3.65
	.004	<.0001	<.0001	.039	ns	.014	.005	<.0001
Employment arrangement (*n* = 2688)								
Permanent (*n* = 2168)	3.67	3.02	4.20	3.34	4.13	3.30	3.66	3.59
Temporary (*n* = 520)	3.71	3.11	4.09	3.36	4.11	3.35	3.71	3.59
	ns	.026	.016	ns	ns	ns	ns	ns
Work shifts (*n* = 2686)								
Day shift (*n* = 975)	3.76	3.34	4.26	3.60	4.24	3.54	3.80	3.77
Rotational shift (*n* = 1711)	3.63	2.87	4.13	3.19	4.06	3.18	3.59	3.49
	<.0001	<.0001	<.0001	<.0001	<.0001	<.0001	<.0001	<.0001
Quality of care (*n* = 2603)								
4–6 (*n* = 163)	2.28	2.24	3.68	2.49	4.01	2.47	2.70	2.87
7-8 (*n* = 1596)	3.62	2.96	4.13	3.26	4.09	3.26	3.60	3.53
9-10 (*n* = 844)	3.96	3.33	4.36	3.64	4.22	3.57	3.98	3.84
	<.0001	<.0001	<.0001	<.0001	<.0001	<.0001	<.0001	<.0001

1: leadership, 2: requiring factors of work, 3: motivating factors of the work, 4: working environment, 5: working welfare, 6: participation in decision making, 7: sense of community; range 1 (lowest) to 5 (highest); ns: not significant.
